# Condomless Anal Sex Associated With Heterogeneous Profiles Of HIV Pre-Exposure Prophylaxis Use and Sexual Activities Among Men Who Have Sex With Men: A Latent Class Analysis Using Sex Diary Data on a Mobile App

**DOI:** 10.2196/33877

**Published:** 2021-12-23

**Authors:** Yi-Fang Yu, Huei-Jiuan Wu, Stephane Wen-Wei Ku, Po-Hsien Huang, Chia-Wen Li, Poyao Huang, Carol Strong

**Affiliations:** 1 Department of Public Health College of Medicine National Cheng Kung University Tainan Taiwan; 2 The Kirby Institute University of New South Wales Sydney Australia; 3 Division of Infectious Diseases Department of Medicine Taipei City Hospital Renai Branch Taipei Taiwan; 4 Department of Psychology National Chengchi University Taipei Taiwan; 5 Center for Infection Control and Department of Internal Medicine National Cheng Kung University Hospital Tainan Taiwan; 6 Institute of Health Behavior and Community Sciences National Taiwan University Taipei Taiwan

**Keywords:** mobile apps, pre-exposure prophylaxis, PrEP, latent class analysis, men who have sex with men, MSM, condom, sex diaries, adherence, app, sex, diary, sexual health, HIV, Taiwan, risk, prevention

## Abstract

**Background:**

New innovative technologies, such as mobile apps, have been developed to increase pre-exposure prophylaxis (PrEP) adherence and the use of log sex diaries. The contiguity of mobile apps reduces the recall bias that generally affects reported condom and PrEP use. However, none of the currently used mobile apps were designed for event-driven PrEP users, and few studies have demonstrated the potential usage of sex diary data to facilitate the understanding of the different HIV risks among heterogeneous profiles of sex diaries and PrEP use.

**Objective:**

We aim to discriminate the heterogeneous profiles of sex events and PrEP use and examine the risk of condomless anal sex among different types of sex events.

**Methods:**

We recruited 35 adult men who have sex with men from two medical centers in Taiwan since May 2020 and followed up for four months. Participants were on PrEP or willing to take PrEP. They were asked to log their sex events, PrEP use, and dosing regimens on a mobile app to improve their PrEP adherence. Latent class analysis was used to distinguish profiles of sex events and PrEP use. Indicators included correct intake of PrEP for each sex event, participants’ sexual positioning, partner’s HIV status, and age.

**Results:**

A total of 551 sex events were classified into three classes by latent class analysis: PrEP nonadherent flip-flopping (234/551, 42%), PrEP imperfect-adherent power bottoming (284/551, 52%), and PrEP adherent serodiscordant topping (33/551, 6%). “PrEP nonadherent flip-flopping” sex events were more likely to involve condomless anal sex than “PrEP imperfect-adherent power bottoming” (OR 1.83, 95% CI 1.03-3.25) after considering random intercepts for individuals, and this class needed to increase their PrEP adherence and use of condoms. “PrEP imperfect-adherent power bottoming” realized their own risk and packaged PrEP with condoms to protect themselves. Up to 99% (32/33) of sex events in “PrEP adherent serodiscordant topping” were protected by PrEP, but all of the sex events in this group were condomless.

**Conclusions:**

Using the sex diary data could advance the capacity to identify high-risk groups. HIV prevention strategy should be more flexible and combine PrEP with condom use for future HIV prevention.

## Introduction

Pre-exposure prophylaxis (PrEP) is an effective tool for HIV prevention [1]. The number of PrEP users has increased in recent years [2]. In Taiwan, the majority of PrEP users are men who have sex with men (MSM) [3], and it is estimated that 8.9% of MSM were on PrEP in 2019 [4]. A question has emerged from the scale-up of PrEP use regarding whether MSM have decreased their intention of using condoms since they are on PrEP. MSM PrEP users may tend to practice condomless anal sex because they perceive PrEP use decreases their risk of HIV infection [5-8]. A few studies did not find support for the increase of condomless anal sex after PrEP initiation [1,9,10]. The evidence of the association between condomless sex and PrEP use remains inconsistent. The practice of condom use needs to be addressed in PrEP implementation because other sexually transmitted infections may increase. Moreover, if PrEP users are stigmatized as those who prefer condomless sex [11], PrEP scalability may be impeded.

To address whether PrEP use is associated with condomless anal sex in MSM requires measurement of condom use during sex and PrEP intake. Most studies have used self-reported surveys to measure condom use and PrEP adherence [12-15], but recall bias affected the accuracy when participants were asked to recall how many times they used condoms or took PrEP over a long period. Some studies asked participants whether they used condoms during the last anal sex event to reduce the recall bias [16-18], but a one-time sex event neither reflects the long-term decision-making of condom use nor assesses the longitudinal change of PrEP adherence. Although self-reporting bias is still inevitable, using a website survey or mobile apps to measure condom and PrEP use can be more immediate than traditional surveys [19-22]. Additionally, PrEP adherence could be measured by drug concentrations in hair or dried blood spots and Wisepill bottle openings [23]. However, drug concentration testing is costly and time-consuming, and Wisepill bottle openings may underestimate PrEP adherence if the bottle is not being used [23]. There has been no perfect measurement for condom and PrEP use until now, but a mobile app that allows users to log their PrEP use and sex diary at any time or place may be more suitable for monitoring HIV risk in MSM. Sex diaries have been used to track sexual encounters, reduce risky sexual behavior [19-21], and facilitate PrEP uptake [19]. Therefore, a sex diary on a mobile app that gathers the information of each sex event, such as condom use and partner’s HIV status, is important for researchers to examine condom use with different sexual partners and track whether or not PrEP is taken correctly.

Because the data from sex diaries contain various characteristics, latent class analysis has usually been used for classifying the characteristics of sexual partners in each sex event to reduce the dimensions of data and create profiles of sexual activities to facilitate outcome analysis. Classifying the patterns of sex events and PrEP use is crucial to identifying which patterns were associated with condomless anal sex. Factors such as a partner’s HIV status and age, sex position, condom use, and dosing regimen of PrEP should be considered in relation to whether one has adhered to PrEP as those factors affect the decision-making of condom use. For example, having sex with an older partner was more likely to involve condomless anal sex [24]. MSM who identified as bottoms were less likely to use condoms than those identified as tops [18]. MSM were more likely to practice condomless anal sex with a partner whose HIV status was the same as themselves, compared with those who had sex with a partner whose HIV status was different [25]. Using mobile apps for MSM to log PrEP uptake and sex diaries, including the risk factors mentioned above, can help researchers identify the subgroups of sex events and their relationship with condom use.

In this study, we used event-level data collected from sex diaries in a mobile app that allowed MSM PrEP users to record their PrEP intake, choice of dosing regimen, and information for each sex event. We aimed to understand whether the decision of condom use in a sex event could be evaluated based on one’s PrEP use and perception of HIV risk. Specifically, we described the pattern of sex diaries and the proportion of correct PrEP use in relation to condom use and the sexual partners’ HIV status. We then used latent class analysis (LCA) to identify heterogeneous sex diaries and PrEP use profiles. Lastly, we examined whether condom use was related to the various sex diaries profiles involving PrEP use. This study demonstrates innovative implications for sex diary data, and the findings will broaden applications in further HIV interventions.

## Methods

### Study Population and Procedure

We recruited participants referred by physicians and PrEP navigators from two medical centers in two major cities of Taiwan (Taipei and Tainan). The following MSM were eligible for inclusion: (1) HIV-negative, (2) age ≥20 years, (3) currently taking PrEP or willing to initiate PrEP after enrollment, (4) had at least 4 episodes of anal sex with men in the previous month, and (5) are willing to install our mobile app—the UPrEPU app—on their smartphone device. Eligible participants interested in this study were informed about the purpose and goal of the study. In the informed consent process, research assistants explained which types of data would be collected by the UPrEPU app. All the data were de-identified, and participants’ personal information was unrecognizable. People who agreed to join the study were asked to provide signed informed consent, and research assistants tutored them regarding the use of the UPrEPU app. Data collected on the app was kept private and confidential. Recruitment began in May 2020, and each participant was followed for 4 months. Participants were tested for HIV at each monthly follow-up. Participants could receive US $20 cash for each follow-up as an incentive. More detailed study procedures were described elsewhere [26].

The UPrEPU was designed to remind the users to take PrEP based on their dosing regimen, potential time, and pre-arranged date for the sex event and monitor their PrEP adherence. More than half of MSM in Taiwan (56%) chose to take PrEP with an event-driven (ED) dosing regimen [27], a regimen that required individuals to take two pills 2 to 24 hours before sex, and followed by taking one pill 24 hours after sex and another pill 48 hours after sex [28]. This is the first app that considered the complexity of the ED dosing regimen, and the reminders for taking ED PrEP were contingent on the timing of sex events. The UPrEPU app allowed users to track whether their sex events were protected by PrEP and log their dynamic PrEP-dosing choices. Participants could also record relevant information regarding each sexual event, including whether a condom was used, participants’ sexual positioning, sexual partner’s age, and HIV status. The present study was approved by the Institutional Review Board of the National Cheng Kung University Hospital (IRB: B-BR-107-076-T).

### Latent Class Indicators

#### Correct Intake of Prep for Each Sex Event

For each sex event, whether or the MSM had correctly used PrEP was based on their dosing regimen and categorized as: (1) correct use of the daily dosing regimen, (2) correct use of the ED dosing regimen, and (3) incorrect use of either dosing regimen. Because taking at least 4 pills per week was suggested to provide enough protective PrEP concentrations [29], correct use of a daily dosing regimen was defined as at least 4 pills taken 168 hours before each sex event. Correct use of an ED dosing regimen was defined as taking 2 pills before and another 2 after each sex event. More specifically, the 2 pills before should be taken 2 to 24 hours before each sex event. However, if at least one pill was taken 25 to 168 hours before each sex event, taking only one pill 2 to 24 hours before each sex event was allowed [28]. The selection of when to take the 2 pills after each sex event was based on when the pill was taken right before sex. Our measurement allowed a 2-hour buffer for the participant to log his PrEP use into the app. Participants could also log their previous PrEP use. For example, if participants took 1 or 2 pills X hours before each sex event (X ranged from 2 to 24), they should take another single pill from 22 to 26 hours after the time X. The last pill should be taken 46 to 50 hours after the time X. Incorrect use referred to those in neither of the above groups.

#### Participants' Sexual Positioning

Participants were asked to report whether they practiced insertive, receptive anal sex, or both during each sex event.

#### Partner’s HIV Status

Response options of each participant’s sex partner’s HIV status consisted of the following: unknown HIV status, negative and on PrEP, negative but not on PrEP, positive with an undetectable viral load (UVL), or positive with an unknown viral load.

#### Partner’s Age

We asked participants to estimate the age of their sexual partner for each event; the options included less than 20, 21 to 30, 31 to 40, 41 to 50, 51 to 60, and above 60 years old. The comparison between the participants’ and their sexual partners’ age was used as the latent class indicator, including “partner was at least one age category younger than I,” “the same as participant’s age category,” and “partner was at least one age category older than I.”

#### Statistical Analysis

The pattern of sex events during 4 months was presented for each participant. For each sex event, we measured whether a condom was used and whether PrEP was taken correctly, along with the choice of the dosing regimen. LCA was conducted using R version 4.0.3 (R Core Team) and the poLCA package [30]. We used LCA to classify sex events into subgroups. Latent class indicators included participants' PrEP dosing regimens during each sex event, their sexual positions, their sexual partner’s HIV status, and the age comparison between participants and their sexual partner. The models were estimated 10 times, with 500 iterations for each run; the solutions with the largest likelihood values were considered maximum likelihood estimates. The model with the lowest Akaike Information Criteria (AIC) [31], lowest Bayesian Information Criteria (BIC) [32], and highest entropy was considered the best-fitting model [33].

Posterior class membership probabilities were used to assign a predicted class to each sex event. Logistic regression analysis was used to compare the difference in condom use between classes. Since one participant could provide more than one sex event, we further used mixed-effects logistic regression with random intercepts for participants.

## Results

### Participants

The study enrolled 35 users who installed the UPrEPU app. We excluded 3 (8.6%) users who did not enter any sex event in the app. The average age of the remaining 32 (91.4%) users was 29.3 years (SD 4.9). During 4 months, no seroconversion was reported. The number of sex events in 4 months ranged from 1 to 66 (mean number: 17.2). A total of 551 sex events were recorded in this study. Table 1 lists the characteristics of sexual partners and sex events. Condoms were used in 22% (123/551) of sex events. Participants reported correct use of ED PrEP in 64% (352/551) of sex events and correct use of daily PrEP in 14% (79/551). Insertive anal sex was practiced in 35% (193/551) of sex events; receptive anal sex was practiced in 54% (295/551); 11% (63/551) of sex events reported practicing both insertive and receptive anal sex in the same sex event. Among the HIV and PrEP status of users’ sexual partners in all events, 38% (209/551) of sexual partners’ HIV status was unknown, 21% (118/551) were on PrEP, 30% were negative and not on PrEP, 11% (59/551) were positive with UVL, and there were no positive partners with unknown viral load. Half of the sexual partners (273/551) were reported in the same age category as the participants.

**Table 1:** Characteristics of sexual partners and sex events (N=551).

Participant characteristics		Values, n (%)
**Whether condom was used**		
	Yes	123 (22)
	No	428 (78)
**Participants’ PrEP^a^ dosing regimens during the sex event**		
	Correct use of daily PrEP	79 (14)
	Correct use of ED^b^ PrEP	352 (64)
	Incorrect use of PrEP	120 (22)
**Participants’ sexual positioning**		
	Insertive anal sex	193 (35)
	Receptive anal sex	295 (54)
	Both insertive and receptive anal sex in the same sex event	63 (11)
**Partner’s HIV status**		
	Unknown HIV status	209 (38)
	Negative and on PrEP	118 (21)
	Negative but not on PrEP	165 (30)
	Positive with an undetectable viral load	59 (11)
	Positive with an unknown viral load	0 (0)
**Partner’s age^c^**		
	Partner was at least one age category younger	154 (28)
	The same as participant’s age category	273 (50)
	Partner was at least one age category older	124 (23)

^a^PrEP: pre-exposure prophylaxis.

^b^ED: event-driven.

^c^Age category difference was based on the following age categorical variables of the sexual partners reported by the participants: less than 20, 21 to 30, 31 to 40, 41 to 50, 51 to 60, and above 60 years old.

**Figure 1:**
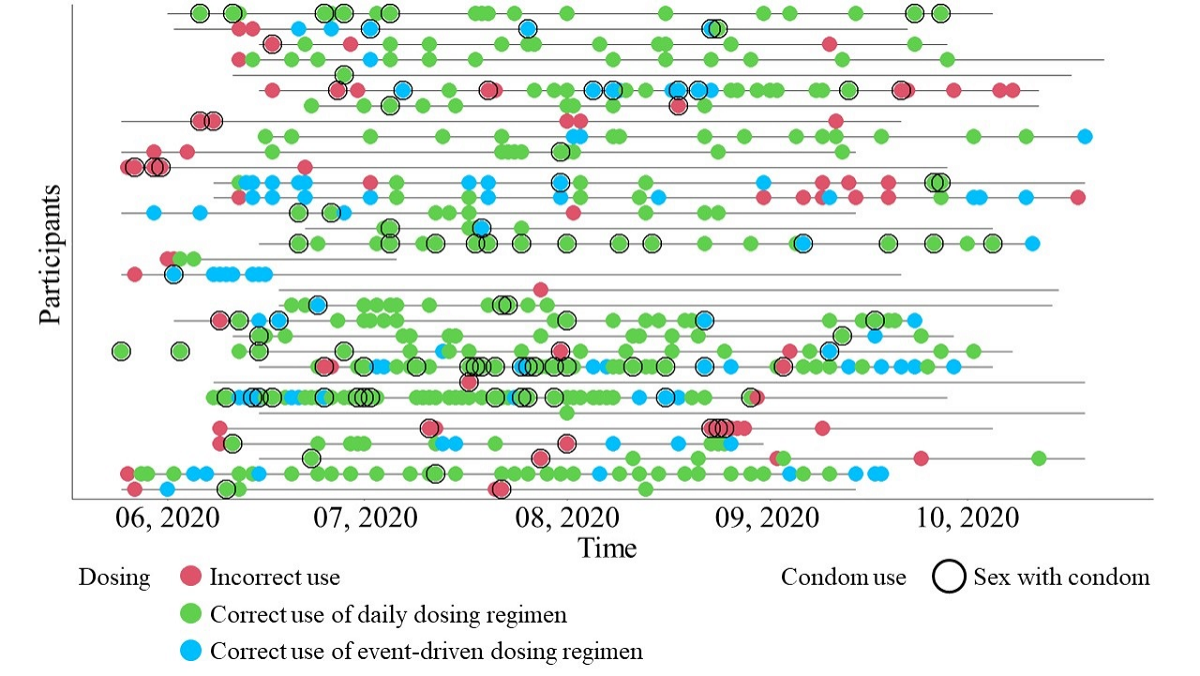
Sex events of each participant with dosing regimen and condom use (N=551).

Among 551 sex events, 83% (455/551) were protected by either PrEP or condoms, 18% (99/551) were protected by both PrEP and condoms, and 17% (96/551) were protected neither by PrEP nor by condoms (Figure 1). Among 32 participants, only one (3.1%) person was protected by PrEP and wore condoms during every sex event. There were 7 (22%) MSM protected by PrEP every time that had at least 1 condomless sex event and 24 (75%) MSM that had at least 1 sex event protected neither by PrEP nor a condom.

### Latent Class Analysis

The statistics of one-class to five-class models are shown in Table 2. Compared with a four-class model, the three-class model had lower BIC. Although the four-class model had lower AIC than three clusters, BIC is more appropriate to indicate the best-fitting model [34]. The three-class model also had the highest entropy. Therefore, we selected the three-class model as the best solution.

**Table 2:** Tests of model fit to identify the optimal number of latent classes.

	Log likelihood	AIC^a^	BIC^b^	Entropy
1 Class	–2306	4629	4668	1.00
2 Class	–2251	4540	4622	0.56
3 Class	–2213	4484	4609	0.76
4 Class	–2187	4453	4621	0.71
5 Class	–2177	4452	4664	0.77

^a^AIC: Akaike Information Criteria.

^b^BIC: Bayesian Information Criteria.

### Characterization of Latent Classes

The conditional probabilities of sex events within each class are listed in Table 3. Class 1 comprised 42% (234/551) of the sex events and was named “PrEP nonadherent flip-flopping*,*” showing the highest proportion of incorrect use of PrEP (45/234, 19.2%), performing both insertive and receptive anal sex in the same sex event (58/234, 24.8%), HIV status of partner unknown 103/234, 44.0%), and partner on PrEP (86/234, 36.8%). This class showed the lowest proportion of having a partner at least one age category older (21/234, 9%). Class 2 had the highest proportion of performing receptive anal sex in sex events (281/284, 98.9%), HIV-negative partners, but not on PrEP (130/584, 45.8%). PrEP protected almost 90% (251/284) of sex events in Class 2. Class 2 comprised 52% (284/551) of sex events and was named “PrEP imperfect-adherent power bottoming.” Class 3 had the highest proportion of correct use of ED PrEP (29/33, 88.0%), HIV-positive partner with UVL (31/33, 94.0%), performing insertive anal sex (33/33, 100%), and having a partner who was at least one age category older (24/33, 72.7%). It also had the lowest proportion of incorrect use of PrEP (1/33, 3.0%), HIV status of partner unknown (2/33, 6.1%), and having a partner who was in the same age category as the participant (0/33, 0%). Class 3 comprised 6% (33/551) of sex events and was named “PrEP adherent serodiscordant topping.”

**Table 3:** Latent classes and conditional probabilities of sex events (N=551).

		PrEP nonadherent flip-flopping (n=234)	PrEP imperfect-adherent power bottoming (n=284)	PrEP adherent serodiscordant topping (n=33)
**Participants' PrEP^a^ dosing regimens during the sex event**				
	Correct use of daily PrEP	0.285	0.165	0.120
	Correct use of ED^b^ PrEP	0.524	0.720	0.867
	Incorrect use of PrEP	0.191	0.114	0.013
**Participants' sexual positioning**				
	Insertive anal sex	0.613	0.011	1.000
	Receptive anal sex	0.139	0.989	0.000
	Both insertive and receptive anal sex in the same sex event	0.248	0.000	0.000
**Partner’s HIV status**				
	Unknown HIV status	0.441	0.363	0.048
	Negative and on PrEP	0.368	0.094	0.000
	Negative but not on PrEP	0.177	0.457	0.000
	Positive with an undetectable viral load	0.014	0.086	0.952
**Partner’s age^c^**				
	Partner was at least one age category older	0.090	0.288	0.746
	The same as participant’s age category	0.563	0.495	0.000
	Partner was at least one age category younger	0.347	0.217	0.254

^a^PrEP: pre-exposure prophylaxis.

^b^ED: event-driven.

^c^Age category difference was based on the following age categorical variables of the sexual partners reported by the participants: less than 20, 21 to 30, 31 to 40, 41 to 50, 51 to 60, and above 60 years old.

### Clusters and Condomless Anal Sex

The proportion of condomless anal sex in each class was 85% (198/234), 69% (197/284), and 100% (33/33), respectively (Table 4). Since the sex events in “PrEP adherent serodiscordant topping” were all condomless, we only compared the probability of condomless anal sex between “PrEP nonadherent flip-flopping” and “PrEP imperfect-adherent power bottoming.” In logistic regression, “PrEP nonadherent flip-flopping” events were 1.43 times more likely to be condomless anal sex compared to “PrEP imperfect-adherent power bottoming” (OR 2.43, 95% CI 1.57-3.76; *P*<.001). After adjusting for the random intercepts, the odds for condomless anal sex were still significantly different between “PrEP nonadherent flip-flopping” and “PrEP imperfect-adherent power bottoming” (OR 1.83, 95% CI 1.03-3.25; *P*=.04).

**Table 4:** Logistic regression for condomless anal intercourse among three classes.

		% of condomless anal sex	OR	95% CI	*P* value
**Model 1**					
	PrEP^a^ nonadherent flip-flopping	85%	2.43	1.57-3.76	<.001
	ref: PrEP imperfect-adherent power bottoming^b^	69%	—	—	—
	PrEP adherent serodiscordant topping^c^	100%	—	—	—
**Model 2**					
	PrEP nonadherent flip-flopping^d^	85%	1.83	1.03-3.25	.04
	ref: PrEP imperfect-adherent power bottoming^b^	69%	—	—	—
	PrEP adherent serodiscordant topping^c^	100%	—	—	—

^a^PrEP: pre-exposure prophylaxis.

^b^PrEP imperfect-adherent power bottoming was the reference group; hence, empty cells were shown in the table.

^c^PrEP adherent serodiscordant topping was not included in logistic regression due to 100% of condomless anal intercourse; hence empty cells were shown in the table.

^d^Adding random intercepts for individuals.

## Discussion

Our study outlined 3 types of users and their behavioral patterns through analyzing their logs. This is the first study to classify event-level data and investigate the relationship between condom use and the heterogeneous profiles of sexual activities, including PrEP uptake among MSM. We demonstrated the use of event-level data of PrEP intake and sex diary logs from a mobile app to capture PrEP adherence in the real world. The sex diary allowed users to self-monitor and researchers to track unprotected sexual behavior and PrEP adherence. Even though high PrEP adherence was observed in our sample, only one-quarter (8/32) of participants had 100% PrEP adherence during the study period. If other HIV prevention methods such as condoms were practiced, imperfect PrEP adherence would not always be a concern; however, if neither PrEP nor condom use is well-adhered, such sexual activities would be exposed to higher HIV risk.

For example, “PrEP nonadherent flip-flopping” required more public health attention because they were neither PrEP nor condom protected. “PrEP nonadherent flip-flopping” comprised both bottoms and tops on a mixed PrEP-dosing regimen with unknown HIV status partners and had significantly lower odds of using condoms versus the “PrEP imperfect-adherent power bottoming” group. One possible reason for not using condoms among the “PrEP nonadherent flip-flopping” group may be that up to one-third (86/234, 36.8%) of sexual partners in this group were HIV-negative and on PrEP. The probability of HIV infection from a partner who was on PrEP was relatively low, and it may have resulted in reduced intention to use condoms [5,6]; however, 44% (103/234) of partners were still HIV-unknown in this group. Even though no participants acquired HIV in the 4-month study period, this group may have been exposed to HIV risk longitudinally. MSM require interventions to assist in taking PrEP correctly, such as reminders or notifications from mobile apps or individualized consultations with their PrEP navigators about their difficulties.

We identified one class, the “PrEP imperfect-adherent power bottoming,” mainly comprised of bottoms with HIV-negative partners, not on PrEP. This group may have been aware of their own risk and therefore practiced HIV prevention behavior: 90% (251/284) of sex events were protected by PrEP, and almost one-third (87/284, 30.6%) were protected by condoms. A discrete choice experiment study used conditional logic analysis to understand the preference for PrEP and condoms based on the hypothesized risk of HIV among gay, bisexual, and other MSM and showed that condoms were used as additional protection besides PrEP when there is a perceived increase in HIV risk [35], which was similar to the behavior of condom use in the “PrEP imperfect-adherent power bottoming.” Whether MSM in this class used condoms depended on their perception of the HIV risk in each sex event. This was also the largest class in our sample, which showed a potential for a combination prevention intervention that adapts to the fluctuating risk for each individual.

The “PrEP adherent serodiscordant topping” class, mainly comprised of tops and partners who were HIV positive with UVL, chose to protect themselves with PrEP instead of condoms—all sex events in this class were condomless. Reasons for MSM to have sex without condoms if they were already on PrEP include reduced fear of HIV, lessened anxiety of having sex due to PrEP use, and maintaining sexual pleasure [6,7]. Furthermore, MSM might decide not to use condoms when having sex with a regular sexual partner due to the mutual trust that friends would not put them at risk [6]. Some MSM indicated that they did not want to use condoms, and therefore they showed strong adherence to PrEP [6]. In this class, we found that PrEP had been successfully implemented, and the concept of “undetectable equals untransmissible” had been well-accepted. Public health researchers still need to stress the importance of using condoms to prevent other sexually transmitted infections. However, with the broadened options for HIV prevention, condoms might not be the first choice for many people in the real world. Therefore, health educational campaigns may need to reprioritize prevention information to achieve optimal effectiveness.

Sex diary data used in this study uniquely included measurement for ED PrEP adherence, such as hours of sex events, instead of only dates. Compared to the apps designed for a daily dosing regimen, the UPrEPU app provided reminders for ED PrEP users to take PrEP based on when sex happened and users’ previous dosing regimens. Adherence to ED PrEP can be calculated only when the hours of the sex event can be logged on an app such as UPrEPU. Such a feature is particularly important since ED PrEP use is more prevalent during the COVID-19 pandemic [36].

The strength of our study was its use of sex diaries to capture the fluctuating HIV risk of sexual behaviors and to classify the diverse patterns of sex events. Most studies have identified the different HIV risks based on the individual level [12-15]. They categorized MSM into different subtypes, but MSM perform various types of sexual behaviors in the real world, which suggests that analyzing event-level data is closer to reality. Identifying the risky class from the diverse patterns of sex events helps researchers build up a specific intervention more intentionally. We offer a more comprehensive recommendation for MSM by looking at different sex roles and PrEP adherence. Based on our findings, future mobile apps can consider giving notifications according to each user’s previous patterns of sex events.

### Limitations

This study has the following limitations. First, participants could see whether they took PrEP correctly in each sex event from the UPrEPU app. This may have interfered with their own judgment for whether they were protected by PrEP and affected their decision to use condoms. Without the app's assistance, PrEP users might not perceive the PrEP adherence correctly, and the decision-making of condom use would be relying solely on their self-perception, which could either be over-estimating or underestimating PrEP adherence.

Second, some contexts of sexual activities were not yet collected in our study. More details of sexual activities may change the categorization in LCA. Information such as whether the partner is a steady sex partner, uses chemsex/sexualized drugs, and belongs to particular sexual networks may be confounders for the association between PrEP adherence and condom use. Lastly, the representative of the study population may be limited due to the recruitment and inclusion criteria. We included participants from only two medical centers and those who had a relatively high frequency of sex events, which restricted the generalizability to MSM who did not use PrEP to protect themselves or whose frequency of sex events was low.

### Conclusions

Our study advances the understanding of the association between HIV risk and the different types of sexual activities. We demonstrated the potential to use mobile app-based sex and PrEP diary for the future development of effective intervention programs by identifying high-risk groups. This study identified that one class, “PrEP nonadherent flip-flopping,” demanded more support for implementing an HIV prevention strategy. “PrEP imperfect-adherent power bottoming” demonstrated that condoms were used as extra protection other than PrEP, and “PrEP adherent serodiscordant topping” showed almost perfect PrEP adherence but risked other sexually transmitted infections. Future HIV interventions should consider combining various strategies to improve the effectiveness of HIV prevention programs.

## Abbreviations

AIC: Akaike Information Criteria
BIC: Bayesian Information Criteria
ED: event-driven
LCA: latent class analysis
MSM: men who have sex with men
PrEP: pre-exposure prophylaxis
UVL: undetectable viral load
